# Urinary tract infection caused by *Edwardsiella tarda*: a report of the first case in Iran

**DOI:** 10.1186/s12879-022-07960-9

**Published:** 2022-12-28

**Authors:** Abolfazl Gilani, Roham Sarmadian, Manijeh Kahbazi, Parsa Yousefichaijan

**Affiliations:** 1 Department of Pediatric Surgery, Tehran University of Medical Sciences, Tehran, Iran; 2 Infectious Disease Research Center (IDRC), Arak University of Medical Sciences, Arak, Iran; 3 Department of Pediatrics, Arak University of Medical Sciences, Arak, Iran

**Keywords:** *Edwardsiella tarda*, Urinary tract infection, Waterborne infection

## Abstract

**Bckground:**

*Edwardsiella tarda*, an Enterobacteriaceae family member, is prevalent in different aquatic settings and rarely infects humans. As a result of eating raw or undercooked seafood, humans become infected through their intestinal tracts. Extraintestinal infections have been reported infrequently, mostly in immunocompromised and chronically ill patients.

**Case presentation:**

Our report describes a case of urinary tract infection caused by *E. tarda* in a 4-year-old female patient with a history of urinary tract infection and a complaint of fever, dysuria, and frequency. *E. tarda* was identified as the pathogen isolated from the urine culture. The patient's symptoms were alleviated after receiving ceftriaxone and then nitrofurantoin.

**Conclusion:**

This case demonstrates that even in immunocompetent patients, *E. tarda* can infect extraintestinal organs, including urinary tract. Our patient represents the first case of *E. tarda* infection in Iran and due to the fact that this pathogen is transmitted by aquatic animals, there is a possibility of infecting more aquatic animals and humans in Iran; therefore, the necessary precautions should be taken.

## Background

*Edwardsiella tarda* is a gram-negative, facultative anaerobe belonging to the Enterobacteraceae family [1]. This bacterium is an unusual human pathogen and has been cultured from the samples of human feces, blood, urine, cerebrospinal fluid, bile, peritoneal fluid, and wounds [2–4]. It is prevalent in water systems and is widely recognized as a pathogen of aquatic organisms [5]. *E. tarda* infection risk factors include exposure to these organisms and consumption of contaminated foods, such as sushi, raw fish, and raw meat [3, 6]. In humans, an infection with *E. tarda* typically manifests as enteritis. However, extra-intestinal infections including soft tissue infections, septicemia, hepatobiliary infections, meningitis, and osteomyelitis have also been reported [7].

Urinary tract infection (UTI) is one of childhood's most common bacterial infections [8]. Enterobacteriaceae families are the most common causes of UTIs. The most frequent organism is *Escherichia coli*, which can cause UTIs in 80 to 90% of children [9]. However, sometimes UTIs can be caused by an atypical microorganism [10]. *E. tarda* is an extremely uncommon UTI cause. *E. tarda* is typically responsible for UTIs in immunocompromised patients and those with a urethral catheter. To date, no cases of *E. tarda*-associated UTIs have been reported in children. In this study, we described a 4-year-old child with prior medical history of recurrent UTI, with the complaint of dysuria and polyuria, and a final diagnosis of UTI caused by the *E. tarda* microorganism.

## Case presentation

A four-year-old girl who had been experiencing fever, frequency, and dysuria for a week was brought to the pediatric clinic. Her parents also mentioned having a headache beginning from 5 days ago. Nausea, vomiting, diarrhea, and abdominal pain were not mentioned. She was first in birth order and was delivered vaginally at 39 gestational weeks. Her birth weight was 3630 g, and measurements of her weight (16 kg), height (98 cm), and head circumference (48 cm) were normal. Her family did not recently have any gastrointestinal or urinary complaints. She was toilet trained but had infrequent voiding. She had no recent history of traveling or playing in rivers or seawater. The mother of the patient mentioned the patient's consumption of salmon over the last month, which did not result in any gastrointestinal issues. A common goldfish was kept in a tank in residence for almost 2 weeks, and it died about a week ago. The patient’s parents stated that she had put her hand into the fish tank several times. The child had been hospitalized twice for UTI since last year. During the second hospitalization, which was 8 months ago, a voiding cystourethrogram was conducted for the patient, and no signs of vesicoureteral reflux were observed.

On arrival, the patient's general condition was good. Her vital signs were heart rate of 98 beats per minute, 115/65 mmHg blood pressure, respiratory rate of 23 breaths per minute, oxygen saturation of 97% in ambient air, and 38.6 °C axillary temperature. During the examinations, no costovertebral tenderness was detected. Neck rigidity was also negative. The examination of the abdomen and genital area revealed no notable findings.

**Fig. 1:**
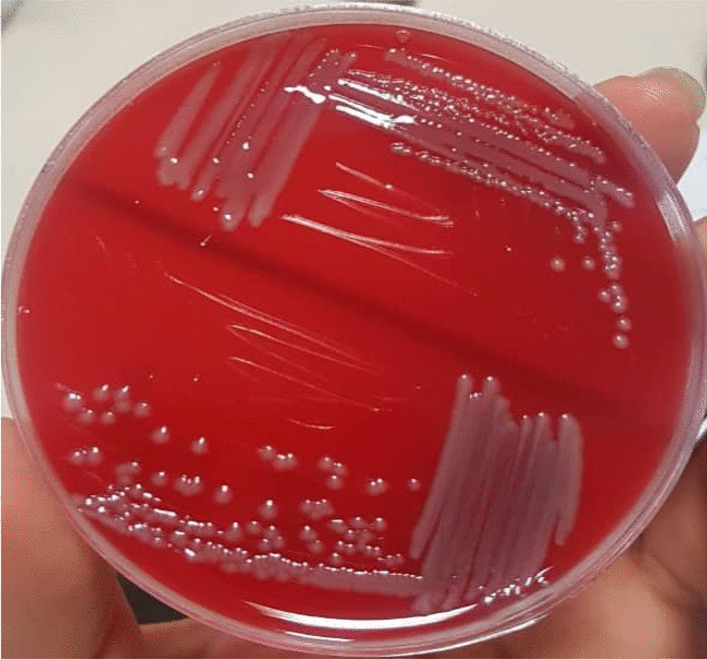
Growth of β hemolytic *E. tarda* colonies on blood agar

**Fig. 2:**
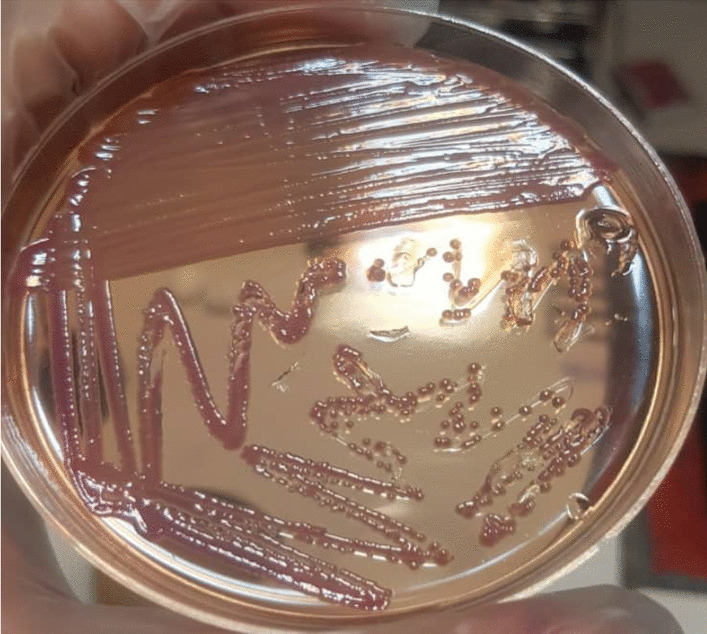
Growth of *E. tarda* colonies on Mac Conkey agar

**Fig. 3:**
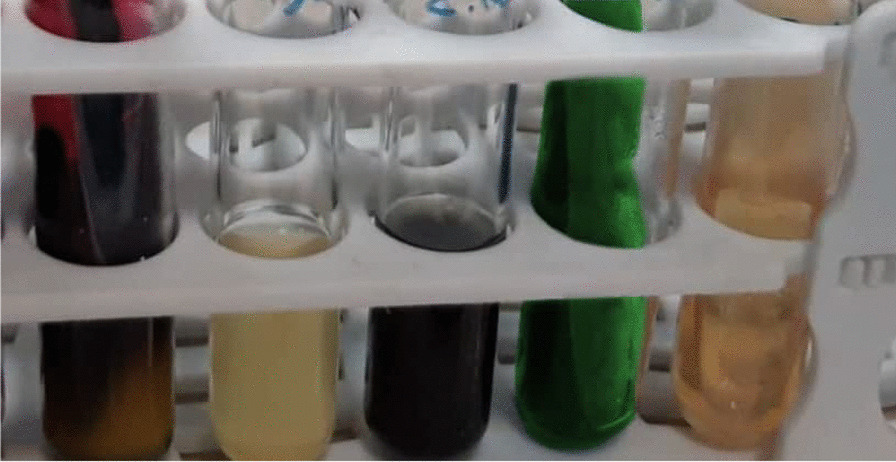
From right to left: negative urease test, negative citrate test, positive sulphide indole motility test (motile and H2S producing), negative VP test, positive Kligler test (H2S producing)

According to a urinalysis performed four days prior at a different center, the patient's urine specimen contained 3–4 red blood cells, many white blood cells, and many bacteria per high-power field, as well as 1+ protein and positive nitritite. Urine culture (UC) had also been performed, and the results revealed *E. tarda* species of more than 100,000 colony-forming units resistant to cefixime, ciprofloxacin, cotrimoxazole, and nalidixic acid, but sensitive to meropenem, tetracycline, and nitrofurantoin. However, the blood culture was negative. According to a later call made to the center's microbiologist for diagnostic information, the specimen was cultured on MacConkey and blood agar containing 5% sheep red blood cell, and it was later determined to be *E. tarda* via chemical tests as follows: The organism was motile and rod-shaped, and hydrogen sulfide production and indole production tests were positive for the organism. Mannitol could not be fermented by the organism, but glucose could. Additionally, the urease, *O*-nitrophenyl-beta-d-galactoside, cytochrome oxidase, citrate, and Vogeus-Proskauer (VP) tests yielded negative results. Figures 1, 2 and 3 illustrate a portion of the urine culture findings.

The patient's parents had been advised to bring their child to the children's hospital for treatment. Prior to admittance, the patient had not been administered any medication. The patient was hospitalized due to her symptoms and the above tests. Initial blood tests revealed a white blood cell count of 11.93 × 10^3^/µL with a differential of 59% neutrophils and 36% lymphocytes, hemoglobin level of 13.32 gr/dL, platelet count of 359 × 10^3^/µL, blood sugar level of 96 mg/dL, and C-reactive protein level of 3+. Due to the patient's headache complaint, which did not improve despite medication use at home, a magnetic resonance imaging of the brain was performed, which indicated no abnormalities.

The patient was administered 600 mg of intravenous ceftriaxone every 12 h along with fluid therapy. On the second day of hospitalization, due to the elimination of fever and improvement in the patient's symptoms, the patient was discharged with the instruction to take nitrofurantoin suspension 25/5 mg, 9 cc every 8 h for 1 week. After a week, the urinalysis results were as follows: 3–4 white blood cells, 0–1 red blood cell, and no bacteria per high power field, and also negative protein and nitrite. The UC revealed no growth after 24 h.

## Discussion and conclusions

*Edwardsiella tarda* is an uncommon pathogen from the Enterobacteriaceae family that can rarely infect humans. The human infection rate is 0.0073% in Japan and 1% in Panama. According to a cohort study conducted in Japan between January 2005 and December 2016, the incidence rate of *E. tarda* is 0.02%, and the mortality rate within 90 days is 26.9% [3, 7, 11].

Clinical complications of *E. tarda* infection are enteritis, liver abscess, cholecystitis, spontaneous bacterial peritonitis, mycotic aneurysm, necrotizing fasciitis, empyema, osteomyelitis, psoas abscess, spinal epidural abscess secondary peritonitis and UTI [12, 13].

Our patient is the first case of human *E. tarda* infection in Iran and the second in the Middle East. According to the reports, *E. tarda* infection cases are mostly reported in East Asia, Australia, North and South America [3].

*Edwardsiella tarda* infection frequently occurs when water temperatures are high, particularly from summer to autumn [14]. It is more common in the elderly, with an average age of 61 years [3]. In contrast, our patient was a 4-year-old child with *E. tarda* infection in spring.

Intestinal symptoms such as gastroenteritis are the most frequent manifestations of *E. tarda* infection [15]. Although *E. tarda* is a rare human pathogen, immunocompromised patients and those with subacute or chronic diseases have a higher incidence of extraintestinal infection [16]. Tamada et al. reported the first case of urosepsis by *E. tarda* in 2009 [12]. The patient was a woman undergoing chemotherapy for advanced uterine cancer. In a study of *E. tarda* infection in Thailand, the organism was isolated from 13 patients with urinary tract infection, none of which were associated with bacteremia, and the majority of patients had chronically indwelling urethral catheters [17]. Our patient, in contrast, had no history of chronic illness or medication or urethral catheter use.

Although *E. tarda* produces β-lactamase, it rarely displays resistance to β-lactam antibiotics [18]. Antibiotics with activity against Gram-negative bacilli, such as the majority of β-lactams, aminoglycosides, tetracyclines, fluoroquinolones, and antifolates, are effective against nearly all *E. tarda* strains [12]. However, in our case, the isolated *E. tarda* strain was resistant to cefixime, cotrimoxazole, and fluoroquinolones. Penicillins, cephalosporins, and carbapenems are frequently used in the treatment of *E. tarda* [3]. Our patient was also initially treated with ceftriaxone, which resulted in the improvement of the patient's symptoms.

Historically, *E. tarda* has been identified in a wide variety of reptiles, amphibians, and fish, including catfish and eels, in both fresh and brackish water settings, and it can also cause disease in these animals [13]. According to the research carried out by Preena et al., *E. tarda* is one of the pathogens that can infect goldfish species and cause 100% mortality in these fish [19]. In addition, this pathogen poses a serious threat to aquaculture globally. Contact with goldfish is likely to cause illness in humans [3]. Likewise, our patient was frequently exposed to goldfish prior to the onset of symptoms. Additionally, the goldfish died after a short time, which could be indicative of infection by *E. tarda*.

Goldfish is one of the 7 Haft Seen items in Iran. Haft Seen is an arrangement of seven symbolic objects that is customarily displayed during the Nowruz holidays beginning on March 21. Goldfish are indigenous to China. According to government accounts, it has been brought from China to Iran in past years due to cultural requirements. In recent years, however, it has been widely cultivated in Iran. Also, each year following the Nowruz holiday, people release millions of these fish into the rivers and lakes of Iran [20]. This is also a significant concern in the United States and Canada. In 2015, for instance, the Canadian government urged people to stop releasing fish into lakes. Considering that goldfish carry several parasites and can reproduce rapidly in harsh environments, they pose a threat to the ecosystem. It is recommended to prevent the release of this type of goldfish in nature. Regular veterinary care is also required for goldfish [21].

This case demonstrates that *E. tarda* can be a urinary pathogen and can induce extraintestinal infection even in individuals with a healthy immune system. Our patient is the first known case of *Edwardsilla* infection in Iran, and since aquatic animals spread this pathogen, there is a likelihood that other humans and aquatic animals will become infected in Iran. As a result, the appropriate safety measures should be adopted, including preventing contact with aquatic organisms and consuming raw or undercooked fish and seafood, as well as isolating fish that may be suspicious or infected from others.

## Data Availability

Data will be provided by the corresponding author on request.

## Abbreviations

*E. tarda*: *Edwardsiella tarda*
UTI: Urinary tract infection
UC: Urine culture
